# Accuracy of Predictive Equations for Metabolizable Energy Compared to Energy Content of Foods for Dogs and Cats Estimated by In Vivo Methods in Brazil

**DOI:** 10.3390/ani15101477

**Published:** 2025-05-20

**Authors:** Pedro Henrique Marchi, Andressa Rodrigues Amaral, Leonardo de Andrade Príncipe, Larissa Wünsche Risolia, Mariana Fragoso Rentas, Ana Beatriz Fasolai, Rafael Vessecchi Amorim Zafalon, Gabriela Luiza Fagundes Finardi, Juliana Toloi Jeremias, Raquel Silveira Pedreira, Júlio Cesar de Carvalho Balieiro, Thiago Henrique Annibale Vendramini

**Affiliations:** 1 Pet Nutrology Research Center (CEPEN Pet), Department of Animal Nutrition and Production, School of Veterinary Medicine and Animal Science, University of Sao Paulo, Pirassununga 13635-000, Brazil; pedro.henrique.marchi@usp.br (P.H.M.); leoprincipe@usp.br (L.d.A.P.); larissa.risolia@gmail.com (L.W.R.); mvmarirentas@gmail.com (M.F.R.); abfasolai@usp.br (A.B.F.); rafael.zafalon@usp.br (R.V.A.Z.); gabriela.finardi@usp.br (G.L.F.F.); balieiro@usp.br (J.C.d.C.B.); 2 Veterinary Nutrology Service, Veterinary Teaching Hospital, School of Veterinary Medicine and Animal Science, University of Sao Paulo, Sao Paulo 05508-270, Brazil; andressa.rodrigues.amaral@usp.br; 3 Grandfood Industria e Comercio (PremieRpet^®^), Nutritional Development Center of PremieRpet, Dourado 13590-000, Brazil; jjeremias@premierpet.com.br (J.T.J.); rpedreira@premierpet.com.br (R.S.P.)

**Keywords:** Brazilian pet food market, calories, cat food, dog food, guaranteed analysis

## Abstract

In small animal nutrition, the caloric content of a diet is expressed as metabolizable energy (ME). The gold standard method for determining this variable is through feeding trials with the target species. However, the high cost and intricacy of this assay lead to the use of indirect estimation methods. The aim of this study was to analyze the main equations employed for estimating ME and compare them with the results of in vivo tests. In total, 451 pet food products in Brazil were evaluated. The ME values were determined via the bromatological values available on labels. The data were analyzed using SAS, and Student’s *t* test was used with a significance of 5%. For all predictive equations, there was a non-conformity between their results and those obtained by in vivo methods. Thus, the prediction equations are only accurate when the exact values of food composition are used, which is hardly applicable since veterinarians and animal owners only have access to the information provided on the labels. Nonetheless, the Atwater system equation proved to be the most reliable for estimating ME, showing the smallest disparity among the evaluated methods. The overall differences between in vivo results and ME estimation with the Atwater system amounted to 3.59% for dry cat foods and −1.94% for dry dog foods. Moreover, although it was also the most accurate for wet foods, the differences were substantially greater (11.99% for cat foods and 8.25% for dog foods). These findings highlight the need for further research to refine ME estimation, which could contribute to improved pet food formulation and help reduce cases of malnutrition in dogs and cats.

## Simple Summary

This study addresses a critical issue in small animal nutrition—accurately estimating the energy content of pet food to support healthy weight management, especially as pet obesity rates rise globally. Traditional methods to measure metabolizable energy—how much energy a pet’s body can actually use—are expensive and not always feasible, as they require live testing with animals. Therefore, various mathematical equations have been developed to estimate this energy, but many of these tools are outdated or do not align well with modern pet food products and labeling practices. In this research, we tested common equations to see how well they matched actual in vivo results. We found that the Atwater equation, despite being older, provided the most reliable estimates for today’s dry and wet pet foods available in Brazil when using the maximum or minimum nutrient values from labels. These findings are valuable for veterinarians and pet food companies, especially those without resources for in vivo tests, and can help improve nutritional recommendations, prevent pet obesity, and reduce malnutrition. Further research could lead to more precise equations, ensuring better nutrition for pets worldwide.

## 1. Introduction

The energy density of a pet food refers to the number of joules or calories present in a given weight or volume [1]. In Brazil, energy density is mainly expressed in kilocalories (kcal) of metabolizable energy (ME) per kg of food [2], which is the amount of energy available to the body considering losses by feces, urine, and expired gases [1]. According to the International System of Units, energy is expressed in units of work (joules), but it can also be expressed in thermodynamic caloric units (calories), based on the caloric value of benzoic acid as a reference standard, which is equal to 4184 joules (0.004184 megajoules) [3]. Metabolizable energy expressed in calories is generally used on the American continent, in contrast to Europe, where the joule is the preferred unit of expression used today. Currently, two methods are used to determine the ME of a pet food: (1) the use of animal experiments, which is considered the gold standard method; (2) the use of predictive equations [4].

Several predictive equations have been proposed aiming to achieve similar outcomes to those observed in animal experiments. The first equation is known as the Atwater factor, and takes into consideration the amount of carbohydrates (CHO), ether extract (EE), and crude protein (CP) of the diet, with ME values of 4.0 kcal/g, 9.0 kcal/g, and 4.0 kcal/g, respectively [5,6]. This equation was later modified to new nutrient energy values (3.5 kcal/g, 8.5 kcal/g, and 3.5 kcal/g for CHO, EE, and CP, respectively) to better estimate the ME value of less digestible products, and it is known as modified Atwater factors [7]. However, since these equations are not as accurate as the gold standard method, the ME result might be under- or overestimated due to several factors that influence digestibility such as ingredient characteristics, food processing, and nutrient interactions with each other, the host, and the gut microbiota [8]. In 2006, the National Research Council (NRC) [1] recommended two new equations for estimating the ME of dog and cat diets, which are considered more accurate. These equations involve a four-step calculation, including the determination of gross energy (GE), energy digestibility, digestible energy (DE), and, finally, ME, with specific adjustments for crude protein and crude fiber (CF).

Later, the European Pet Food Industry Federation (FEDIAF) also adopted the NRC equations for determining the energy content of pet food [9]. This decision was supported by a study by Calvez et al. [10], which compared the precision of the modified Atwater method and the NRC-recommended equations with in vivo ME measurements. Calvez et al. [10] demonstrated that the NRC equations [1] offer a more accurate estimate of ME than the modified Atwater method for dry pet food. For wet food, however, both the modified Atwater method and NRC equations yield moderate accuracy in estimating ME for dogs and cats. According to FEDIAF [9], these findings also contributed to the development of the European standard EN 16967 [11], which references predictive equations for calculating and declaring ME in pet food.

The caloric content of a diet is a key factor in determining the amount of food that should be provided. However, ME values are not always listed on pet food labels, requiring veterinarians to estimate ME using predictive equations [12], based on available information, such as the maximum or minimum levels of each nutrient provided on the label. Considering the evolution of pet food products, the introduction of more digestible ingredients, and increased product diversity, the role of accurate ME estimation in daily pet nutrition has become increasingly significant. Additionally, it is essential to evaluate the applicability of predictive equations across different regions worldwide, given the variation in commercial products. Therefore, this study aimed to analyze the equations available in the literature and assess their reliability for predicting ME under these new conditions.

## 2. Materials and Methods

### 2.1. Commercial Products

A total of 667 diets were selected, including 408 extruded dry food diets (208 for dogs and 125 for cats). Of these, 17 dog diets were discarded (11 for providing only the ME estimate and 6 due to insufficient information), while 59 cat diets were excluded (50 for providing only the ME estimate and 9 due to insufficient information). For the wet food diets, 148 were selected (71 for dogs and 77 for cats). Among these, 12 dog diets were discarded (9 for providing only the ME estimate and 3 due to insufficient information), along with 17 cat diets (13 for providing only the ME estimate and 4 due to insufficient information).

**Figure 1:**
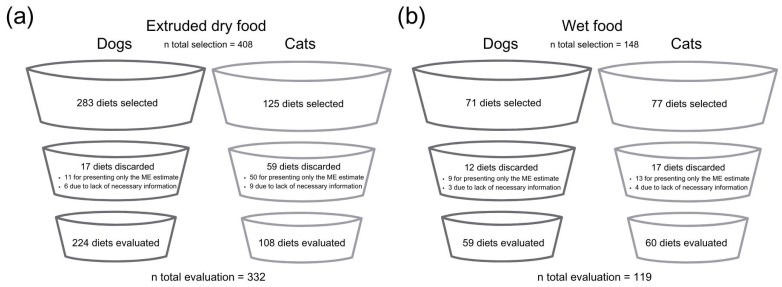
A diagrammatic representation of the number of diets selected, excluded, and evaluated in the study: (**a**) Extruded dry diets for both dogs and cats; (**b**) wet diets for both dogs and cats.

In total, 451 diets were included in the final analysis—332 extruded dry food diets (224 for dogs and 108 for cats) and 119 wet food diets (59 for dogs and 60 for cats) (Figure 1). To obtain detailed product information, 26 pet food companies were contacted. According to FEDIAF [9], food was classified as wet if it had a moisture content of 60% or higher (e.g., canned or pouched products). Only ME values obtained through animal experiments were accepted for inclusion in this study, regardless if the company used the method of total fecal collection (with or without urine collection), or indicators, according to AAFCO [4] guidelines.

The percentages of moisture, CP, EE, CF, and ash described on the label were considered. Only CF was considered for the calculations; thus, labels reporting fiber content using other methods, such as total dietary fiber, were excluded. For nutrients whose lack could lead to deficiencies, the minimum limits (protein and fat) were used, and for the others, where the excess addition tends to compromise quality, the maximum limits (moisture, CF, and ash) were used. Nitrogen-free extract (NFE), considered non-structural carbohydrates (sugar, starch, and pectin), was calculated using the equation:

NFE (%) = 100% − (moisture% + CP% + EE% + CF% + ash%).

The average chemical composition of evaluated diets is shown in Table 1.

### 2.2. Metabolizable Energy Estimation Equations

The ME of the diets obtained through in vivo methods were compared to those obtained by the three following equations:

1. Atwater factors [6]:

ME (kcal/kg) = (4.0 × g CP/kg) + (9.0 × g EE/kg) + (4.0 × g NFE/kg),

2. Modified Atwater factors [7]:

ME (kcal/kg) = (3.5 × g CP/kg) + (8.5 × g EE/kg) + (3.5 × g NFE/kg),

3. ME-predictive equations from NRC and FEDIAF [1,9]:
  (a) Determination of GE:
    GE (Kcal/Kg) = (5.7 × g CP/kg) + (9.4 × g EE/kg) + [4.1 × (g NFE/kg + g CF/kg)],
    considering the composition of CP, EE, NFE, and CF in natural matter (NM).
  (b) Percentage energy digestibility (PED):
    PED for cat food = 87.9 − (0.88 × %CF, in DM),
    PED for dog food = 91.2 − (1.43 × %CF, in DM).
  (c) Determination of DE:
    DE (kcal/kg) = GE × (PED/100).
  (d) Determination of ME:
    ME for cat food (kcal/kg) = DE − (0.77 × g CP/kg),
    ME for dog food (kcal/kg) = DE − (1.04 × g CP/kg),

where joules were used as the unit of measurement described, respecting the conversion of 1 calorie to 4184 joules (0.004184 megajoules).

### 2.3. Statistical Analysis

All data were analyzed using the Statistical Analysis System, version 9.4 (SAS Institute Inc., Cary, NC, USA). Initially, the normality of residuals and the homogeneity of variances were assessed using the Shapiro–Wilk and Student’s *t*-tests, respectively. To compare the reference method (in vivo testing) with the predictive equations, ANOVA and orthogonal contrasts were applied. The contrasts evaluated were C1 (in vivo testing vs. Atwater factors [6]), C2 (in vivo testing vs. modified Atwater factors [7]), and C3 (in vivo testing vs. NRC [1]/FEDIAF [9]). A significance level of *p* < 0.05 was used for all of the tests.

## 3. Results

### 3.1. Dry Extruded Diets

**Figure 2:**
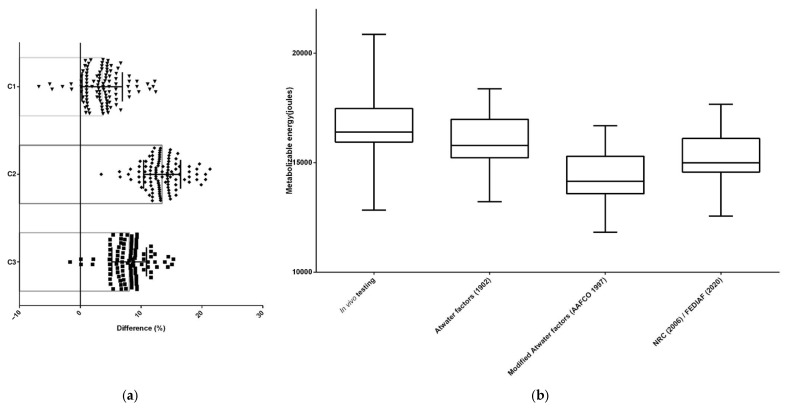
The differences between methodologies for predicting and estimating the ME of dry extruded cat foods in the Brazilian market (*n* = 108); (**a**) the differences identified by orthogonal contrasts [C = contrast; C1—in vivo testing (reference) × Atwater factors; C2—in vivo testing (reference) × modified Atwater factors; C3—in vivo testing (reference) × NRC/FEDIAF]; (**b**) the differences between metabolizable energy estimates [1,6,7,9].

**Figure 3:**
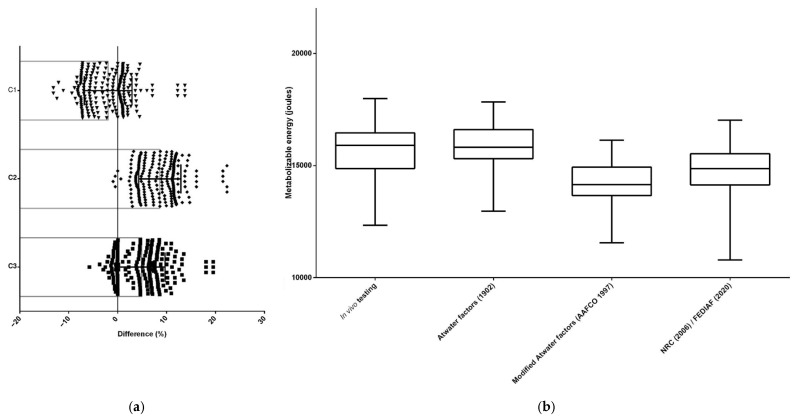
The differences between methodologies for predicting and estimating the ME of dry extruded dog foods in the Brazilian market (*n* = 224); (**a**) the differences identified by orthogonal contrasts [C = contrast; C1—in vivo testing (reference) × Atwater factors; C2—in vivo testing (reference) × modified Atwater factors; C3—in vivo testing (reference) × NRC/FEDIAF]; (**b**) the differences between metabolizable energy estimates [1,6,7,9].

For all dry extruded diets (*n* = 332), the ME estimated through predictive equations differed (*p* < 0.05) from the ME determined by in vivo testing. The Atwater factors [6] equation resulted in the smallest difference (%) between these two methods and, therefore, was the most accurate equation to estimate the ME of dry extruded cat food (*n* = 108) (Figure 2 and Table 2) and dry extruded dog food (*n* = 224) (Figure 3 and Table 3).

### 3.2. Wet Foods

**Figure 4:**
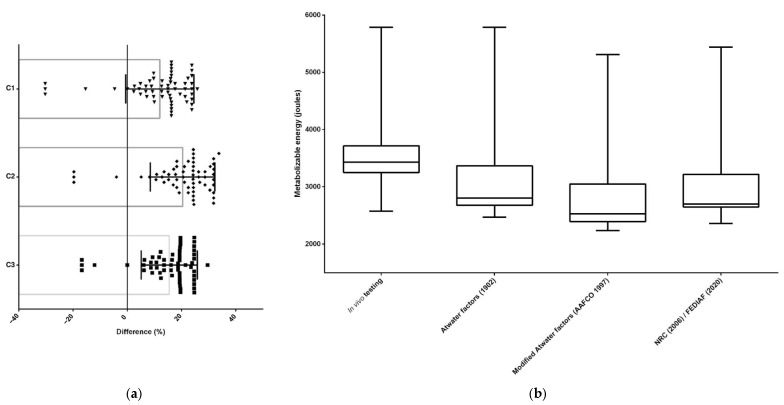
The differences between methodologies for predicting and estimating the ME of cat wet foods in the Brazilian market (*n* = 60); (**a**) the differences identified by orthogonal contrasts [C = contrast; C1—in vivo testing (reference) × Atwater factors; C2—in vivo testing (reference) × modified Atwater factors; C3—in vivo testing (reference) × NRC/FEDIAF]; (**b**) the differences between metabolizable energy estimates [1,6,7,9].

**Figure 5:**
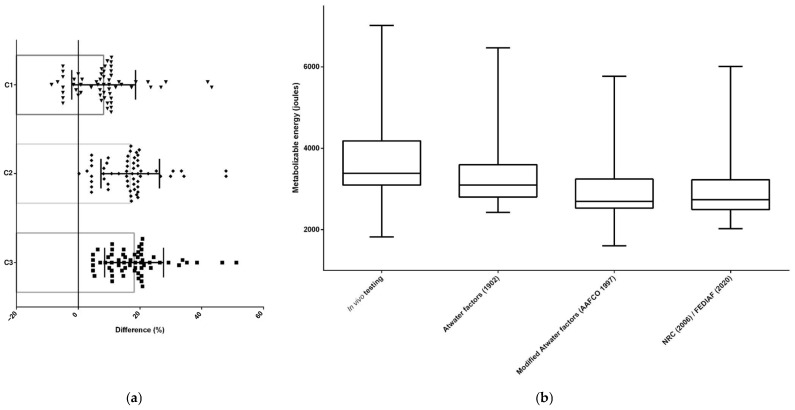
The differences between methodologies for predicting and estimating the ME of wet dog foods in the Brazilian market (*n* = 59); (**a**) the differences identified by orthogonal contrasts [C = contrast; C1—in vivo testing (reference) × Atwater factors; C2—in vivo testing (reference) × modified Atwater factors; C3—in vivo testing (reference) × NRC/FEDIAF]; (**b**) the differences between metabolizable energy estimates [1,6,7,9].

The ME results obtained for the wet foods (*n* = 119) were also different using ME-predictive equations in contrast with ME determination through in vivo testing, for both cats (*n* = 60) (Figure 4 and Table 4) and dogs (*n* = 59) (Figure 5 and Table 5). As with dry foods, the Atwater factors [6] resulted in the smallest differences (%) compared to modified Atwater and NRC/FEDIAF equations; therefore, this is the most accurate equation to estimate the ME of wet cat and dog foods.

## 4. Discussion

The Atwater factors equation resulted in more precise values of ME for both dry extruded and wet food compared to other equations. This result differs from those verified by Castrillo et al. [3], Hall et al. [13], and Calvez et al. [10] since for Castrillo et al. [3] the most accurate predictive equations were the NRC and FEDIAF equations, followed by the modified Atwater factors equation. However, the authors emphasized that both equations underestimate and overestimate ME for diets with low and high fiber content, respectively. Hall et al. [13] conducted a retrospective analysis of 331 digestibility studies in dogs and 227 in cats. They found that for both dry and wet foods, the most accurate equation for predicting ME was the modified Atwater factors equation for dog food, while both the modified Atwater factors and NRC/FEDIAF equations were most accurate for cat food. The authors attributed the differences in values obtained from the NRC and FEDIAF equations for dog food, as well as the Atwater factors equation [6] for both dog and cat foods, to variations in food digestibility. Specifically, the reduced digestibility of wet foods led to an overestimation of ME when using the NRC/FEDIAF and Atwater equations; conversely, the more digestible dry foods resulted in a slight underestimation of ME with these same equations [13].

Calvez et al. [10] also reported different results after comparing equations and concluded that ME estimation using the modified Atwater factors equation was worse compared to the NRC and FEDIAF equations. However, some limitations should be considered in their study, since some data were incomplete, limiting the number of available food trials to be analyzed. Additionally, they performed these studies with a small number of animals from a single research colony and a limited number of feeding trials were conducted using wet food. Additionally, these authors observed that when using measured GE (without subtracting energy lost in fermentation, urine, and feces) rather than the predicted GE, the NRC and FEDIAF equations performed better. Although this is an interesting finding, it is not easily achieved in normal routine, since the GE measurement is performed using a calorimeter, which is not easily available to veterinarians and in veterinary clinics. Thus, an alternative for the future is to stimulate pet food companies to also include the food’s GE value on the label.

Additionally, it is worth mentioning that the differences in the results of the current study may be associated with the sample size used. In the present study, the analyzed products were only those available in the Brazilian market. Thus, in relation to extruded dry foods, the underestimated values of ME can be due to inaccurate nutrient concentrations used in the equation, since the information on nutritional composition may not accurately reflect the chemical composition of the tested products. This can be explained by the fact that the regulation of commercial pet food is provided by Normative Instruction number 09, published in 2003 [2], and establishes that manufacturers are required to include minimum percentages of CP and EE, and maximum percentages for moisture, ash, and crude fiber [2].

The objective of this study is precisely to demonstrate the practical reality of applying predictive equations. It is well established that the most current prediction equations are more accurate when applied to the exact composition of the evaluated products; however, this is rarely the case. Kienzle et al. [14] conducted a trial to develop an equation based on the analysis of GE by bomb calorimetry and the prediction of apparent energy digestibility through the fiber content of the diet. The results showed a slight overestimation of ME values when compared to the gold standard method, which supported the proposal of a new equation. This equation aims to mitigate potential variation caused by GE estimation and is far more applicable in practice than in vivo methods. Nonetheless, in practice, ME estimates are often based solely on label composition, which relies on minimum and maximum guaranteed values. For instance, comparisons between label values and laboratory bromatological analyses in North America have shown that commercial pet foods typically contain, on average, 1% more EE and CP than the minimum guaranteed, and 1% less moisture and ash than the maximum declared values [15]. Currently, such comparative studies are not available for pet foods outside the North American market, but they may prove valuable for improving ME estimation globally.

Furthermore, Sutherland et al. [16] recently evaluated the equations used to develop commercial feeding guidelines (traditional Atwater equation or modified Atwater equation) in 200 dry dog foods sold in Canada. Their findings revealed that 38% of the evaluated diets did not calculate ME based on AAFCO recommendations. This highlights a critical issue—pet food labels may not always provide ME values derived from standardized or accurate methods, making it difficult for veterinarians and pet owners to rely solely on this information. Given these inconsistencies, having a reliable and easy-to-use predictive tool is essential for veterinary professionals to make more accurate dietary recommendations, ensuring optimal nutrition for companion animals.

Discrepancies between ME values obtained from animal experiments and those predicted by equations are expected. However, it is crucial to identify an equation that more accurately reflects the nutrient composition and digestibility profile of modern pet food formulations. Existing predictive equations were likely developed based on ingredient profiles and formulations that have since evolved, potentially leading to inaccuracies in current estimations. As pet food manufacturing continues to incorporate novel ingredients and processing techniques, there is a growing need to refine or develop new predictive models that align more precisely with these changes. By improving the accuracy of ME estimation, it may be possible to enhance diet formulation, optimize nutrient utilization, and ultimately contribute to better health outcomes for companion animals. The Atwater factors equation is the oldest and was initially used for products with very high digestibility, and also takes into consideration the same values of nutrients (minimum); however, it resulted in the best prediction of ME. This fact also emphasizes the hypothesis of an increase in the digestibility and improvement of ingredients used in the pet food industry in recent years, when compared to the composition and digestibility of ingredients used in the 1970s, 1980s, 1990s, or even 2000s. Still, it may be that the errors compensate for each other; i.e., that the overestimate of digestibility compensates for the underestimate of nutrients by the minimum levels labeling. That may be helpful in practical nutrition in countries with the corresponding maximum and minimum labeling legislation.

Regarding wet foods, it is worth noting that their macronutrients and energy content differ from those of extruded dry foods [1]. This can result in a difference in accuracy between the different equations studied. Most wet foods are formulated with a relatively small proportion of NFE, resulting in levels of digestible CHO between 4% and 13% [17] since they do not require CHO for the process of starch gelatinization during extrusion. These types of foods also have high inclusion levels of fresh or frozen meat, which have a high protein and fat content [17]. As different predictive equations can have distinct results when applied to wet or dry foods, it is preferable to analyze the data separately [10]. In addition, these different results are expected, since most of the predictive equations [6,7] were determined before the consumption of wet food became popular [8] and, therefore, the equations were not developed for this food profile.

It is important to emphasize that veterinarians and pet owners only have access to the information provided on food labels, which includes maximum and minimum nutrient levels, as stipulated by current legislation. This limitation means that the ME values calculated using the equations evaluated in this study can be easily utilized and applied in daily practice, highlighting the need for more reliable predictive equations to support this routine. Moreover, the most commonly used equations tend to underestimate ME values. These equations either align with Brazilian legislation [2], which allows for ME prediction using the modified Atwater factors equation, or they adhere to the primary bibliographic references utilized by professionals in the field of nutrition [9,18].

Depending on the quality of the diets (super premium, premium, or economic), the difference between ME-predictive values obtained through these equations and the real ME value can be higher than 20%. The practical consequence of these underestimated calculations is that if the amount of food consumed by any animal is determined based on the ME of the food [1,9] this will result in an excess of the ingested amount and, consequently, in weight gain and obesity [10,18]. The underestimated values of a diet’s ME may be one of the main factors resulting in a high incidence of the obesity presently evident, or at least a contributing factor. This problem becomes more important considering that even though pet owners look for veterinarian orientation to give the correct amount of food recommended to their pets, the amount of food may be inappropriate due to the inaccuracy of the ME-predictive equations used, as pointed out in the present study.

Another point to be highlighted is the wide variation in the composition of the wet foods evaluated; this large variation makes it difficult to estimate any of the prediction equations already proposed. Still, as a reference to the composition, some evaluated products present a crude fiber content greater than 8% of the dry matter, and according to the NRC [1], this estimate may be inaccurate. Considering that none of the equations currently available truly expressed a similarity with in vivo tests, it would be interesting to find new formulas to better estimate pet food ME. Then, these new formulas should be applied to current pet food products and might be specific to each type of product, such as grain-free, light, or wet foods, in order to reduce major chemical composition differences. These measures are important since an equation only fits properly to a food if it takes into consideration the characteristics of this food [1]. Another important quality control for an equation would be to use different datasets to generate and to test it, ensuring that it is accurate with different samples from the same market segment.

Nevertheless, it should be emphasized that even though future ME equations have become effective, there will always be the challenge of the animal’s intrinsic variations that can alter energy utilization and ME value, such as age, sex, neutered status, husbandry, and activity level [19,20]. Considering this, owners and professionals must always adjust caloric intake according to individual responses, aiming to maintain an ideal body condition score for each case. This is even more important in weight loss programs for patients with overweight or obese. In these situations, simply reducing calories through a maintenance diet will lead to undernutrition due to the fixed formula. Cutting calories reduces not only overall food intake but also essential nutrients such as proteins, vitamins, fats, and minerals. The use of fortified diets is essential to prevent this issue, and their ME should be determined by the manufacturer through in vivo assays to ensure reliable values for the calculation of both calorie and nutrient intake during the process.

By comparing the differences in energy intake between the NRC equations for inactive/castrated or active/intact dogs (95 kcal × body weight^0.75^ versus 110 kcal × body weight^0.75^) and cats (75 kcal × body weight^0.67^ versus 100 kcal × body weight^0.67^), the change in energy intake categories differs by 13.63% for dogs and 25% for cats. Therefore, the authors of this work believe that differences between the equations should be, at least, inferior to these percentages to avoid significant changes in energy recommendations for each category.

## 5. Conclusions

In conclusion, the Atwater factors equation, even though it is the oldest equation, resulted in the best ME prediction for nutritional practice and routine for dry extruded and wet dog and cat food products currently commercially available in Brazil, evaluating the nutrient composition of the label with its maximum or minimum levels depending on the nutrient. This raises new perspectives on the prediction of pet food ME and demonstrates that further studies are needed in order to improve this area of knowledge. Therefore, with these improvements, it will be possible to reduce cases of malnutrition in dogs and cats in countries where the label legislation allows for the inclusion of maximum or minimum content values.

## Figures and Tables

**Table 1:** Chemical composition of natural matter in pet foods commercialized in Brazil (*n* = 451) [mean ± standard deviation (minimum–maximum)] in g/kg of food.

Nutrient	Extruded		Wet	
	Cats (*n* = 108)	Dogs (*n* = 224)	Cats (*n* = 60)	Dogs (*n* = 59)
Moisture (maximum)	93.9 ± 9.46 (80.0–120.0)	99.9 ± 8.39 (90.0–120.0)	816.2 ± 20.74 (750.0–840.0)	807.3 ± 38.99 (630.0–860.0)
Crude protein (minimum)	346.8 ± 45.69 (250.0–500.0)	262.9 ± 31.74 (200.0–355.0)	92.7 ± 13.94 (70.0–125.0)	80.3 ± 11.63 (34.0–95.0)
Fat (minimum)	141.4 ± 40.84 (80.0–230.0)	131.5 ± 32.89 (70.0–211.0)	35.2 ± 15.68 (20.0–95.0)	39.2 ± 10.23 (20.0–70.0)
Crude fiber (maximum)	44.2 ± 20.87 (18.0–150.0)	40.4 ± 20.27 (19.0–150.0)	17.1 ± 4.56 (6.0–25.0)	18.5 ± 4.34 (6.0–30.0)
Ash (maximum)	81.2 ± 7.51 (53.0–105.0)	76.4 ± 15.50 (6.0–110.00)	26.1 ± 4.84 (13.0–33.0)	25.1 ± 6.93 (14.3–38.0)
Nitrogen-free extract	292.5 ± 59.56 (145.0–435.0)	388.9 ± 55.35 (240.0–485.0)	12.7 ± 13.10 (0.0–56.2)	29.6 ± 43.17 (0.0–227.0)

**Table 2:** Metabolizable energy estimation and the difference between methodologies determining the metabolizable energy of dry extruded cat foods in the Brazilian market (*n* = 108) (mean ± standard deviation).

Variables	Predictive Equations				P ^1^		
	In Vivo Testing (Reference)	Atwater Factors [6]	Modified Atwater Factors [7]	NRC and FEDIAF [1,9]	C1	C2	C3
Metabolizable energy (MJ/kg)	1.66 ± 0.13	1.60 ± 0.11	1.44 ± 0.11	1.53 ± 0.10	<0.0001	<0.0001	<0.0001
Difference ^2^ (%)	—	3.59 ± 3.32	13.45 ± 3.05	8.05 ± 2.84	—	—	—

Legend: C = contrast; ^1^ C1—in vivo testing (reference) × Atwater factors; C2—in vivo testing (reference) × modified Atwater factors; C3—in vivo testing (reference) × NRC/FEDIAF; ^2^ differences between ME results obtained through animal experiment and ME obtained through predictive equations.

**Table 3:** Metabolizable energy estimation and the difference between methodologies for determining the metabolizable energy of dry extruded dog food in the Brazilian market (*n* = 224) (mean ± standard deviation).

Variables	Predictive Equations				P ^1^		
	In Vivo Testing (Reference)	Atwater Factors [6]	Modified Atwater Factors [7]	NRC and FEDIAF [1,9]	C1	C2	C3
Metabolizable energy (MJ/kg)	1.56 ± 0.12	1.59 ± 0.09	1.42 ± 0.09	1.48 ± 0.10	0.0070	<0.0001	<0.0001
Difference ^2^ (%)	—	−1.94 ± 4.87	8.62 ± 4.29	4.90 ± 4.79	—	—	—

Legend: C = contrast; ^1^ C1—in vivo testing (reference) × Atwater factors; C2—in vivo testing (reference) × modified Atwater factors; C3—in vivo testing (reference) × NRC/FEDIAF; ^2^ the differences between ME results obtained through animal experiments and ME obtained through predictive equations.

**Table 4:** Metabolizable energy estimation and the differences between methodologies for determining the metabolizable energy of wet cat foods in the Brazilian market (*n* = 60) (mean ± standard deviation).

Variables	Predictive Equations				P ^1^		
	In Vivo Testing (Reference)	Atwater Factors [6]	Modified Atwater Factors [7]	NRC and FEDIAF [1,9]	C1	C2	C3
Metabolizable energy (MJ/kg)	0.36 ± 0.06	0.31 ± 0.06	0.28 ± 0.06	0.29 ± 0.06	<0.0001	<0.0001	<0.0001
Difference ^2^ (%)	—	11.99 ± 12.56	20.41 ± 11.90	15.45 ± 10.34	—	—	—

Legend: C = contrast; ^1^ C1—in vivo testing (reference) × Atwater factors; C2—in vivo testing (reference) × modified Atwater factors; C3—in vivo testing (reference) × NRC/FEDIAF; ^2^ the differences between ME results obtained through animal experiments and ME obtained through predictive equations.

**Table 5:** Metabolizable energy estimation and the differences between methodologies for determining the metabolizable energy of wet dog foods in the Brazilian market (*n* = 59) (mean ± standard deviation).

Variables	Predictive Equations				P ^1^		
	In Vivo Testing (Reference)	Atwater Factors [6]	Modified Atwater Factors [7]	NRC and FEDIAF [1,9]	C1	C2	C3
Metabolizable energy (MJ/kg)	0.36 ± 0.09	0.33 ± 0.08	0.30 ± 0.07	0.29 ± 0.07	0.0370	<0.0001	<0.0001
Difference ^2^ (%)	—	8.25 ± 10.34	16.85 ± 9.48	18.12 ± 9.55	—	—	—

Legend: C = contrast; ^1^ C1—in vivo testing (reference) × Atwater factors; C2—in vivo testing (reference) × modified Atwater factors; C3—in vivo testing (reference) × NRC/FEDIAF; ^2^ the differences between ME results obtained through animal experiments and ME obtained through predictive equations.

## Data Availability

The original contributions presented in this study are included in the article. Further inquiries can be directed to the corresponding author.
